# Supplementary material for: Strategies to Overcome Resistance to Immune-Based Therapies in Osteosarcoma
Source: Int J Mol Sci. 2023 Jan 2;24(1):799. doi: 10.3390/ijms24010799 (PMC9821333; doi:10.3390/ijms24010799)
Supplement: Supplementary file 1 [file ijms-24-00799-s001.zip › ijms-2031338-supplementary.pdf]

Table S1. List of clinical trials involving immunomodulating agents or approaches in high-grade osteosarcoma (HGOS), which are either presently active or that have been concluded in the last 6 months without providing published results. In the "Indication" column, only the eligibility criteria for HGOS patients have been reported. Trials with available results have been described in the text.

**Number referred in Figure 3**	**Compound/Therapeutic strategy**	**Structure**	**Mechanism of action**	**Indication**<br><br>**ClinicalTrials.gov NCT identifier (protocol acronym)**<br><br>**Participating Countries**	**Stage of development**<br><br>**(time period)**
**1**	**Mifamurtide** combined with post-operative chemotherapy	muramyl tripeptide linked to dipalmitoyl-phosphatidyl-ethanolamine to facilitate incorporation into liposomes	macrophage stimulant used with conventional drugs	HGOS metastatic at diagnosis or localized HGOS with poor histological response<br><br>NCT03643133 (SARCOME 13)<br><br>France	phase II<br><br>recruiting<br><br>no available results<br><br>(10/2018 - 10/2028)
**2**	**Avelumab** (Bavencio®)	fully human monoclonal antibody against PD-L1	targeting PD-L1	recurrent or progressive HGOS<br><br>NCT03006848<br><br>USA	phase II<br><br>active, not recruiting<br><br>no available results<br><br>(02/2017-01/2023)
**3**	**ZKAB001** (STI-1014; STI-	fully human monoclonal	targeting PD-L1	recurrent or refractory	phase I/II

	A1014)	antibody against PD-L1		HGOS<br><br>NCT03676985<br><br>China	recruiting<br><br>no available results<br><br>(10/2018 - 06/2023)
**Number referred in Figure 3**	**Compound/Therapeutic strategy**	**Structure**	**Mechanism of action**	**Indication**<br><br>**ClinicalTrials.gov NCT identifier**<br>**(protocol acronym)**<br><br>**Participating Countries**	**Stage of development**<br><br>**(time period)**
**4**	**ZKAB001** maintenance therapy after adjuvant chemotherapy	anti-PD-L1 antibody	targeting PD-L1	HGOS<br><br>NCT04359550<br><br>China	phase III<br><br>not yet recruiting<br><br>no available results<br><br>(06/2020 - 06/2023)
**5**	**Nivolumab** (Opdivo®) plus **Sunitinib** (Sutent®, previously known as SU11248) after standard treatment	Nivolumab: monoclonal antibody against PD-1<br><br>Sunitinib: RTK inhibitor	targeting PD-1 and RTK	advanced soft-tissue and bone sarcomas<br><br>NCT03277924<br>(GEIS-52)<br><br>Spain, Italy	phase I/II<br><br>recruiting<br><br>no available results<br><br>(05/2017 - 09/2022)
**6**	**Nivolumab** (Opdivo®) and **Ipilimumab** (Yervoy®) plus cryotherapy	Nivolumab: monoclonal antibody against PD-1	targeting PD-1 (nivolumab) and CTLA-4 (ipilimumab)	Relapsed/refractory osteosarcoma	phase II<br><br>recruiting

		Ipilimumab: monoclonal antibody against CTLA-4		NCT05302921<br><br>USA	no available results<br><br>(02/2022 – 07/2025)
**7**	**Nivolumab** (Opdivo®) or **Nivolumab** (Opdivo®) and **Azacitidine**	Nivolumab: monoclonal antibody against PD-1	targeting PD-1	recurrent, resectable HGOS<br><br>NCT03628209<br><br>USA	phase I/II<br><br>recruiting<br><br>no available results<br><br>(10/2019 - 07/2022)

Number referred in Figure 3	Compound/Therapeutic strategy	Structure	Mechanism of action	Indication<br><br>ClinicalTrials.gov NCT identifier<br>(protocol acronym)<br><br>Participating Countries	Stage of development<br><br>(time period)
**8**	**Nivolumab** (Opdivo®) and **Regorafenib**	Nivolumab: monoclonal antibody against PD-1	targeting PD-1	recurrent, metastatic HGOS<br><br>NCT04803877 (SARC038)<br><br>USA	phase II<br><br>recruiting<br><br>no available results<br><br>(06/2021 - 06/2026)
**9**	Combination of **Pembrolizumab** and **Cabozantinib**	Pembrolizumab: humanized IgG4 antibody directed against human cell surface receptor PD-1<br><br>Cabozantinib: multi-kinase inhibitor	potential immune checkpoint inhibitory and antineoplastic activities; blocking of PD-1 binding to its ligands, which results in the activation of T-cell-mediated immune responses against tumor cells	advanced HGOS<br><br>NCT05182164 (PEMBROCABOSARC)<br><br>France	phase II<br><br>recruiting<br><br>no available results<br><br>(04/2022 - 10/2025)
**10**	**Atezolizumab** and **Cabozantinib** for the treatment of adolescents and young adults	Atezolizumab: monoclonal antibody against PD-L1<br><br>Cabozantinib: multi-kinase inhibitor	targeting PD-L1	locally advanced, metastatic, recurrent, refractory, or unresectable HGOS<br><br>NCT05019703	phase II<br><br>active, not recruiting<br><br>no available results

				(TACOS)<br><br>USA	(06/2022 - 12/2027)

Number referred in Figure 3	Compound/Therapeutic strategy	Structure	Mechanism of action	Indication<br><br>ClinicalTrials.gov NCT identifier<br>(protocol acronym)<br><br>Participating Countries	Stage of development<br><br>(time period)
**11**	**Camrelizumab** in combination with neoadjuvant chemotherapy	Camrelizumab: humanized anti-PD1 IgG4 monoclonal antibody	targeting PD-1	locally resectable HGOS<br><br>NCT04294511<br><br>China	phase II<br><br>recruiting<br><br>no available results<br><br>(12/2019 - 09/2023)
**12**	**LN-145** or **LN-145-S1** after **Ipilimumab, Nivolumab,** cyclophosphamide IV and fludarabine IV and before Aldesleukin IV and **Nivolumab**	LN-145 is made by collecting Growing specialized white blood cells (T-cells) that are collected from the patient's tumor<br><br>LN-145-S1 is made using a modified process that chooses a specific portion of the T-cells<br><br>Ipilimumab: monoclonal antibody against CTLA-4<br>Nivolumab: monoclonal antibody against PD-1	T cells should specific targeting and killing of tumor cells<br><br>targeting PD-1and CTLA-4	relapsed or refractory HGOS<br><br>NCT03449108<br><br>USA	phase II<br><br>recruiting<br><br>no available results<br><br>(04/2018 - 06/2024)
**13**	**Tislelizumab** in association	Human IgG4 monoclonal	targeting PD-1 in	bone metastatic cancers	phase II

	with chemotherapy	antibody against PD-1 combined with chemotherapy	association with chemotherapy	(including HGOS)<br><br>NCT05241132<br><br>China	recruiting<br><br>no available results<br><br>(11/2021 – 10/2024)
**Number referred in Figure 3**	**Compound/Therapeutic strategy**	**Structure**	**Mechanism of action**	**Indication**<br><br>**ClinicalTrials.gov NCT identifier**<br>**(protocol acronym)**<br><br>**Participating Countries**	**Stage of development**<br><br>**(time period)**
**14**	**Niraparib** and **dostarlimab**	Niraparib: PARP inhibitor with potential antineoplastic activity<br><br>Dostarlimab: PD-1 inhibitor	inhibition of PARP and PD-1	solid tumors (including HGOS)<br><br>NCT04544995<br><br>France, Spain, UK	phase I<br><br>recruiting<br><br>no available results<br><br>(10/2020 – 03/2030)
**15**	**Durvalumab** and **Oleclumab**	Oleclumab: Monoclonal Antibody MEDI9447 that inhibits the ectonucleotidase CD73 (also known as 5-nucleotidase)<br><br>Durvalumab: Human Monoclonal antibody that blocks the interaction of PD-L1 with PD-1 and CD80 inhibiting the immune	help of the body's immune system in killing cancer cells<br><br>interference with the ability of tumor cells to grow and spread	metastatic , recurrent or refractory HGOS<br><br>NCT04668300 (DOSa)<br><br>USA	phase II<br><br>recruiting<br><br>no available results<br><br>(11/2020 - 06/2024)

		evading mechanisms			
16	**Durvalumab and tremelimumab**	Durvalumab: anti-PD-L1 IgG1monoclonal antibody<br><br>Tremelimumab: anti-CTLA-4 human monoclonal antibody	targeting PD-L1 and CTLA-4	advanced or metastatic HGOS<br><br>NCT02815995<br><br>USA	phase II<br><br>recruiting<br><br>no available results<br><br>(08/2016 - 12/2022)

**Number referred in Figure 3**	**Compound/Therapeutic strategy**	**Structure**	**Mechanism of action**	**Indication**<br><br>**ClinicalTrials.gov NCT identifier**<br>**(protocol acronym)**<br><br>**Participating Countries**	**Stage of development**<br><br>**(time period)**
**17**	Humanized **anti-GD2 monoclonal antibody 3F8 (Hu3F8)** combined with GM-CSF	humanized anti-GD2 monoclonal antibody	targeting GD2 with the humanized antibody Hu3F8	recurrent HGOS<br><br>NCT02502786<br><br>USA	phase II<br><br>recruiting<br><br>no available results<br><br>(07/2015 - 07/2023)
**18**	**Dinutuximab** and **Magrolimab**	Dinutuximab beta: IgG1 chimeric monoclonal antibody against GD2<br><br>Magrolimab: A human monoclonal anti-CD47 antibody for the treatment of non-Hodgkin's lymphoma	targeting GD2 and CD47	recurrent or resectable HGOS<br><br>NCT04751383<br><br>USA	phase I<br><br>recruiting<br><br>no available results<br><br>(04/2021 - 01/2024)
**19**	**Pepinemab (VX15/2503)**	anti-SEMA4D monoclonal antibody	immunomodulation	recurrent or refractory HGOS and malignant solid tumors<br><br>NCT03320330<br><br>USA	phase I/II<br><br>active, not recruiting<br><br>no available results

					(01/2018 - 12/2025)

Number referred in Figure 3	Compound/Therapeutic strategy	Structure	Mechanism of action	Indication<br><br>ClinicalTrials.gov NCT identifier<br>(protocol acronym)<br><br>Participating Countries	Stage of development<br><br>(time period)
**20**	**PLX3397** plus **Sirolimus**	PLX3397: inhibitor of of macrophage colony-stimulating factor receptor<br><br>Sirolimus: immunosuppressive agent	PLX3397: selective inhibition of macrophages<br><br>Sirolimus: immunosuppression	unresectable sarcomas (including HGOS)<br><br>NCT02584647<br><br>USA	phase I/II<br><br>recruiting<br><br>no available results<br><br>(11/2015 - 03/2024)
**21**	**Autologous tumour infiltrating lymphocytes** in association with cyclophosphamide and fludarabine	cellular immunological anticancer therapy	Novel immunotherapy based on tumour-infiltrating lymphocytes in patients non-responding to standard treatments	several solid tumors, including recurrent HGOS<br><br>NCT03610490<br><br>USA	phase II<br><br>active, not recruiting<br><br>no available results<br><br>(08/2018- 01/2025)
**22**	**Tumor infiltrating lymphocytes** plus **anti-CTLA-4** and **anti-PD-L1/L2**	Autologous tumor infiltrating lymphocytes after Ipilimumab and followed by Nivolumab	Cellular immunological anticancer therapy plus anti-CTLA-4 and anti PD-L1 and PD-L2 treatment	relapsed or refractory HGOS<br><br>NCT03449108<br><br>USA	phase II<br><br>recruiting<br><br>no available results<br><br>(04/2018 - 06/2024)

Number referred in Figure 3	Compound/Therapeutic strategy	Structure	Mechanism of action	Indication<br><br>ClinicalTrials.gov NCT identifier<br>(protocol acronym)<br><br>Participating Countries	Stage of development<br><br>(time period)
**23**	**Haploidentical transplant and donor NK cells**	cellular and adoptive immunotherapy	HLA-haploidentical hematopoietic cell transplantation followed by an early, post-transplant infusion of donor NK cells will influence the development of particular NK and T cell subtypes	HGOS<br><br>NCT02100891<br>(STIR)<br><br>USA	phase II<br><br>not recruiting<br><br>no available results<br><br>(03/2014 - 12/2022)
**24**	**Transplantation with haploidentical donor peripheral blood stem cells** in conjunction with **Zoledronate**	cellular and adoptive immunotherapy	enhancement of tumor killing by combining transplantation with immunostimulating activity of zolendronic acid	pediatric patients with relapsed or refractory hematologic malignancies or high risk solid tumors, including HGOS<br><br>NCT02508038<br><br>USA	phase I<br><br>recruiting<br><br>no available results<br><br>(02-2016-11/2025)
**25**	Aerosolized **Aldesleukin**	Aldesleukin (also called Proleukin and recombinant human interleukin-2) is a form of interleukin-2 that is	Aldesleukin acts as immunomodulating agent, increasing the activity and growth of	metastatic osteosarcoma, stage IV HGOS<br><br>NCT01590069	phase I<br><br>not recruiting

		made in the laboratory.	white blood cells called T lymphocytes and B lymphocytes, which may help the immune system kill cancer cells	USA	no available results<br><br>(06/2012- 12/2022)
**Number referred in Figure 3**	**Compound/Therapeutic strategy**	**Structure**	**Mechanism of action**	**Indication**<br><br>**ClinicalTrials.gov NCT identifier (protocol acronym)**<br><br>**Participating Countries**	**Stage of development**<br><br>**(time period)**
**26**	**CRX100** combined with oncolytic virus CDSR	*ex vivo* generation of autologous CIK	anti-tumor activity of autologous CIK cells	relapsed or refractory HGOS<br><br>NCT04282044<br><br>USA	phase I<br><br>recruiting<br><br>no available results<br><br>(01/2021 - 03/2025)
**27**	**Dendritic Cell** and tumor cell vaccine	patient-derived dendritic cells vaccine (a type of white blood cells that helps fight infections obtained by leukapheresis)<br><br>tumor cell vaccine: tumor cell lysate	dendritic cells vaccine followed by tumor cell vaccine with or without gemcitabine prior to dendritic cells vaccination	bone sarcoma<br><br>NCT01803152<br><br>USA	phase I<br><br>not recruiting<br><br>no available results<br><br>(01/2014- 07/2024)
**28**	4th generation safety-engineered **CAR T cells** targeting sarcomas	4th generation safety-engineered CAR T cells	CAR-IgT cells targeting sarcoma surface antigens	osteoid sarcomas, including HGOS	phase I/II<br><br>recruiting

				NCT03356782<br><br>China	no available results<br><br>(12/2017 - 12/2023)

Number referred in Figure 3	Compound/Therapeutic strategy	Structure	Mechanism of action	Indication<br><br>ClinicalTrials.gov NCT identifier<br>(protocol acronym)<br><br>Participating Countries	Stage of development<br><br>(time period)
**29**	**EGFR806 CAR T cell** immunotherapy	second generation EGFR-specific CAR T cells	administration of T cells derived from the participant's blood, which have been genetically modified to express either the EGFR receptor alone (EGFR806CAR(2G)-EGFRt) or in addition also the CD19 receptor (CD19CAR(2G)-T2A-HER2tG)	recurrent or refractory solid tumors, including HGOS<br><br>NCT03618381<br><br>USA	phase I<br><br>recruiting<br><br>no available results<br><br>(06/2019 - 06/2038)
**30**	**NY-ESO-1-specific CAR T cells**	CAR T cells targeting NY-ESO-1 positive cells	TCR affinity enhancing specific T cell therapy	bone sarcomas (including HGOS) stage IV or after failure of first-line treatment<br><br>NCT03462316<br><br>China	phase I<br><br>recruiting<br><br>no available results<br><br>(05/2018 - 02/2023)

Number referred in Figure 3	Compound/Therapeutic strategy	Structure	Mechanism of action	Indication<br><br>ClinicalTrials.gov NCT identifier<br>(protocol acronym)<br><br>Participating Countries	Stage of development<br><br>(time period)
**31**	Feasibility and safety study of **fluorescein-specific (FITC-E2) CAR T cells** in combination with parenterally administered folate-fluorescein (UB-TT170)	ex-vivo expanded autologous T cells genetically modified to express an antiFL(FITC-E2) CAR	evaluation of the pharmacokinetics of UB-TT170 in combination with the anti-FL(FITC-E2) CAR T cells	recurrent, refractory HGOS<br><br>NCT05312411<br><br>USA	phase I<br><br>recruiting<br><br>no available results<br><br>(05/2022 - 04/2040)
**32**	**CAR T cells targeting GD2** with interleukin-15 plus iCaspase 9 and Cyclophosphamide	CAR T cells	targeting GD2	relapsed or refractory HGOS<br><br>NCT03721068<br><br>USA	phase I<br><br>recruiting<br><br>no available results<br><br>(02/2019 - 06/2039)
**33**	**GD2-targeted modified T-cells (GD2CART)** and Cyclophosphamide	GD2-targeted CAR T-cells	immune cell therapy based on GD2-CAR-expressing autologous T-lymphocytes (GD2CART)	relapsed, refractory HGOS<br><br>NCT04539366<br>(GD2-CAR PERSIST)<br><br>USA	phase I<br><br>recruiting<br><br>no available results<br><br>(06/2021 - 08/2024)

Number referred in Figure 3	Compound/Therapeutic strategy	Structure	Mechanism of action	Indication<br><br>ClinicalTrials.gov NCT identifier<br>(protocol acronym)<br><br>Participating Countries	Stage of development<br><br>(time period)
**34**	**Anti-GD2 CAR T cells**	GD2-targeting CAR T cells (GD2-CART01)	single infusion of GD2-CART01	recurrent GD2 positive HGOS<br><br>NCT03373097<br><br>Italy	phase I/II<br><br>recruiting<br><br>no available results<br><br>(01/2018 - 01/2027)
**35**	**C7R-GD2 CAR T cells** with or without cyclophosphamide and fludarabine	CAR T cells targeting GD2	cellular immunological anticancer therapy, targeting GD2<br><br>C7R gives the cells a constant supply of cytokine and helps them to survive for a longer period of time	relapsed HGOS<br><br>NCT03635632<br><br>USA	phase I<br><br>recruiting<br><br>no available results<br><br>(04/2019 – 12/2037)
**36**	**iC9-GD2-CAR-VZV-CTLs**	CAR consisting of an anti-GD2 antibody called 14g2a (GD2-CAR), containing parts of the CD28 and OX40 genes, which can stimulate T cells to make them live	treatment with GD2-T cells (also called iC9-GD2-CAR-VZV-CTLs) in combination with a varicella zoster vaccine and	refractory or metastatic GD2-positive HGOS<br><br>NCT01953900<br>(VEGAS)	phase I<br><br>active, not recruiting<br><br>no available results

		longer	lymphodepleting chemotherapy	USA	04/2014 - 10/2034)

Number referred in Figure 3	Compound/Therapeutic strategy	Structure	Mechanism of action	Indication<br><br>ClinicalTrials.gov NCT identifier<br>(protocol acronym)<br><br>Participating Countries	Stage of development<br><br>(time period)
**37**	**CD276-targeted CAR T** administered intravenous and local tumor injection	CAR T cells targeting CD276	targeting CD276, which is highly expressed on the surface of HGOS cells but not in normal peripheral cells or tissues	HGOS<br><br>NCT04864821<br><br>China	early phase I<br><br>not yet recruiting<br><br>no available results<br><br>(05/2021 - 05/2023)
**38**	Multiple **sarcoma-specific CAR-T cells** and sarcoma vaccines	CAR-T cells against GD2, PSMA, Her2, CD276 or other markers and vaccines	multiple CAR T cells with low dose chemotherapy followed by maintenance sarcoma vaccines	relapsed or late stage Osteoid sarcoma<br><br>NCT04433221<br><br>China	phase I/II<br><br>recruiting<br><br>no available results<br><br>(07/2020 - 12/2023)
**39**	**B7-H3-CAR T** cells in association with cyclophosphamide, MESNA and fludarabine	cellular immunological anticancer therapy	CAR T cells targeting B7-H3 antigen	HGOS<br><br>NCT04897321<br><br>USA	phase I<br><br>recruiting<br><br>no available results<br><br>(06/2022 – 03/2027)

Number referred in Figure 3	Compound/Therapeutic strategy	Structure	Mechanism of action	Indication<br><br>ClinicalTrials.gov NCT identifier<br>(protocol acronym)<br><br>Participating Countries	Stage of development<br><br>(time period)
**40**	**B7-H3-CAR T** cells	cellular immunological anticancer therapy	CAR T cells targeting B7-H3 antigen	recurrent/refractory HGOS<br><br>NCT04483778<br><br>USA	phase I<br><br>recruiting<br><br>no available results<br><br>(07/2020 – 12/2040)

Legend: B7H3 or CD276: CD276 molecule; CAR: Chimeric antigen receptor; CIK: Cytokine induced killer cells; CTLA4: Cytotoxic T-Lymphocyte Associated Protein 4; EGFR: Epithelial growth factor receptor; GD2: Disialoganglioside 2; Her2 (ERBB2): Erb-B2 receptor tyrosine kinase 2; NK: Natural killer; PARP: Poly ADP-ribose polymerase; PD-1: Programmed cell death-1; PD-1L, PD-L1: Programmed death-ligand 1; PD-2L, PD-L2: Programmed death-ligand 2; PSMA: Prostate-specific membrane antigen; RTK: Tyrosine kinase; SEMA4D: Semaphorin 4D; TAMs: Tumor associated macrophages.
